# Compositional Dynamics of Gastrointestinal Tract Microbiomes Associated with Dietary Transition and Feeding Cessation in Lake Sturgeon Larvae

**DOI:** 10.3390/microorganisms10091872

**Published:** 2022-09-19

**Authors:** Shairah Abdul Razak, Shaley Valentine, Terence Marsh, John Bauman, Norfarhan Mohd-Assaad, Kim T. Scribner

**Affiliations:** 1 Department of Fisheries & Wildlife, Michigan State University, East Lansing, MI 48824, USA; 2 Department of Applied Physics, Faculty of Science & Technology, Universiti Kebangsaan Malaysia, Bangi 43600, Malaysia; 3 Department of Microbiology and Molecular Genetics, Michigan State University, East Lansing, MI 48824, USA; 4 Institute of Systems Biology, Universiti Kebangsaan Malaysia, Bangi 43600, Malaysia; 5 Department of Integrative Biology, Michigan State University, East Lansing, MI 48824, USA

**Keywords:** aquaculture, diet transition, feeding cessation, gut microbiome, high-throughput sequencing, lake sturgeon, microbiome, protease, dysbiosis

## Abstract

Compromised nutritional conditions associated with dietary transitions and feeding cessation in the wild and during fish aquaculture operations are common and can impact growth and survival. These effects are especially prevalent during early ontogenetic stages. We quantified phenotypic and GI tract microbial community responses with an emphasis on protease-producing bacteria of lake sturgeon (*Acipenser fulvescens*) larvae, a species of aquacultural and conservational importance. To quantify responses associated with experimental food transition and feeding cessation, we performed a 36-day feeding experiment using two treatments: control and diet transition. However, larvae in the diet transition treatment failed to undergo transition and ceased feeding. Larvae in the diet transition treatment exhibited lower growth (total length and body weight) and survival than control larvae. Treatment had a greater effect than ontogenetic changes on taxonomic composition and diversity of the GI tract microbial community. Proteobacteria dominated the GI tract microbial community of the diet transition larvae whereas Firmicutes dominated the GI tracts of control larvae. Most of the 98 identified protease-producing isolates in both treatments were from genera *Pseudomonas* and *Aeromonas*: taxonomic groups that include known fish pathogens. Overall, failing to transition diets affected responses in growth and GI tract microbiome composition and diversity, with the later dysbiosis being an indicator of morbidity and mortality in larval lake sturgeon. Thus, microbiological interrogations can characterize responses to dietary regimes. The results can inform fish culturalists and microbiologists of the importance of dietary practices consistent with the establishment and maintenance of healthy GI tract microbiota and optimal growth during early ontogeny.

## 1. Introduction

Phenotypic and physiological development in vertebrates occurs rapidly during early life stages leading to sequential changes in resource requirements [1]. As organisms age, physiological requirements change, the means used to acquire resources change, and individuals alter resource use including habitats occupied and prey selectivity [2,3]. Changes in foraging abilities enable individuals to capture and consume progressively larger prey, leading to size-influenced dietary changes [4]. For example, many fish species exhibit carnivorous feeding behavior throughout their lifespan but sequentially shift from eating zooplankton to invertebrates to fish [5]. These rapid changes in resource use pose challenges in artificial settings such as aquacultural operations. Throughout early ontogeny, aquaculture professionals attempt to accommodate both fish and hatchery resource needs including dietary requirements and nutritional needs to maximize fish growth and fish health [6].

High mortality associated with the period of first larval feeding acts as a major demographic bottleneck in both wild and cultured organisms [7,8,9]. Mortalities result from larval feeding challenges including rapid physiological and ecological changes and shifts from endogenous to exogenous nutrition sources [10]. During the larval stage, physiological adaptations for the efficient breakdown and absorption of essential nutrients, including appropriate digestive enzymes and absorptive surface areas, are diverse [11,12]. For larvae to grow properly, nutrients such as amino acid, lipids, carbohydrates, vitamins, and minerals are essential for health performance, maximal growth, and survival [13]. Additionally, larvae are particularly sensitive to food availability [7]. Previous studies indicate that larvae receiving an inadequate food source or experiencing an imbalance in nutrients often grow poorly and experience high mortality [10].

High mortality is not conducive to aquaculture production, so aquaculturalists use multiple methods to increase the acceptance of feed during larval periods and ontogenetic resource transitions, thus improving the growth and survival of reared organisms. First, live organisms such as brine shrimp (*Artemia salina*), rotifers, chironomids, or other zooplanktonic animals are commonly fed [14,15,16,17]. These organisms may stimulate the feeding response, and contain many of the essential nutrients for larval fish development [10,18]. Second, feeding regimes including co-feeding or food transitioning may help deliver balanced nutritional regimes, resulting in improved larval growth and survival especially during resource transition periods. These alternative feeding regimens have been used with numerous fish species [13,19,20]; however, issues in providing suitable nutrients for optimal growth persist [20,21,22,23,24]. In extreme cases, larvae may fail to transition to or co-feed on a different diet. When this failure continues, larvae may cease feeding and/or reach the “point of no return” where they cannot recover from a lack of nutrients even if food is available [25,26]. While the growth and survival of larval fishes in aquaculture associated with food sources and amount are widely described [13,27], less well understood are how changes to or the disruption of nutritional succession affect gut physiology and ecology of the organism, including the microbial community.

Previous studies show the GI tract microbial community composition can affect the physiology of an organism [28]. First, functional redundancy in GI tract microbial communities are important [29] and therefore additions and subtractions of community members frequently do not change community function. Second, shifts in the functional composition of the GI tract community composition suggest changes in gut physiological status [30]. Third, dietary induced changes in GI tract microbial composition can have significant effects on metabolism [31]. Finally, feed quantity and composition and subsequently the nutrient environment are strongly tied to gastrointestinal (GI) tract microbiota composition in fish [32,33]. Thus, diet and gut microbiota interactively affect one another and the health of the host.

The presence of healthy GI tract microbiota is essential for normal fish growth and development [34]. However, disruption of feeding regimes and nutrient availability might alter GI tract microbial composition. For example, fish that failed to convert to a different food source during a transition phase and experience food deprivation undergo a phase that represents an ‘energy crisis’ to themselves as well as to the microbiota in the GI tract due to the absence or reduction in the availability of nutrients [35,36]. Findings based on well-studied mammalian (e.g., human) GI tract microbiomes have been extensively documented during periods of health and associated with diseases [37]. Complexities underlying the relationships between the gut microbiota and taxa on the aquatic realm are less well understood. Further research is warranted to ascertain what microbial taxa and fish physiological and morphological features change in relative abundance during feed transitions and feeding cessation.

Sturgeon (family Acipenseridae) are an important fish group of conservation concern in natural habitats and are widely produced in commercial and conservation aquaculture worldwide. In North America, Lake Sturgeon (*Acipenser fulvescens*) is an imperiled species in the Laurentian Great Lakes Region of North America [38]. Populations are supplemented through conservation aquaculture programs by stocking hatchery-reared juveniles [39]. Hatchery rearing of lake sturgeon presents difficulties similar to most aquaculture species such as high mortality during early ontogenetic stages due in part to poor feeding performance, particularly the ability to transition to different feeds during early ontogenetic stages [40,41,42,43]. Diet transitions are routine in lake sturgeon aquaculture practices [44], but may lead to high mortality if larvae do not transition to the newly offered diet [43]. Currently, experimental data are lacking for lake sturgeon growth performance as well as effects of the diet transition on GI tract microbial community composition and diversity during periods of dietary transition or feeding cessation.

The main objective of this study was to document the composition and diversity of GI tract microbial taxa during early ontogenetic stages associated with feeding transition and feeding cessation, and to quantify fish growth and survival. Specifically, we compared GI tract microbial community composition and diversity among larvae raised using different diets and quantified the functional roles of GI tract microbiota, including potentially mutualistic species such as proteolytic bacteria that are involved in the fermentation and production of secondary metabolites [45]. We hypothesized that GI tract microbial community composition of lake sturgeon before, during, and after diet transitions will differ relative to controls (i.e., larval lake sturgeon fed under status quo feeding conditions). The documentation of GI tract microbial communities, specifically microbial taxonomic signatures of community response to feeding cessation associated with ontogenetic dietary transitions and deteriorating fish health generally can be useful to fish managers and expand findings to less well characterized vertebrate systems for microbiologists.

## 2. Materials and Methods

### 2.1. Study Site

The experiment was conducted at the Black River Sturgeon Rearing Facility managed by the Michigan Department of Natural Resources (MDNR) and Michigan State University (MSU) in Onaway, MI, USA, using MSU approved Institutional Animal Care and Use Committee (IACUC) protocols 03/14-037-99 (Hatchery Protocols), 03/14-038-99 (Netting Adults), 03/14-039-99 (Spawning Protocol).

Environmental conditions were monitored throughout the duration of the experiment. Water supplied to hatching jars and aquaria was taken from the Upper Black River and filtered using 50-micron sock filters. Water temperature was monitored hourly using a YSI ProODO Optical DO-Temp meter (YSI, Inc., Yellow Springs, OH, USA). Average daily water temperature ranged from 11.8 °C to 19.7 °C, with the mean (±SD) of 16.4 ± 1.8 °C over the duration of the study. The mean (±SD) flow rate for each 3.0-L aquaria was approximately 440 mL/min (8.8 aquaria cycles/hour). A 9 h light, 15 h dark cycle was maintained using fluorescent lights.

### 2.2. Study Fish, Fish Husbandry, and Feeding Experiment

Lake sturgeon larvae were produced from one adult female and one adult male lake sturgeon from gametes collected during the 2015 sturgeon-spawning season on 3 May from the Upper Black River, in Cheboygan County, MI, USA. Full-sib individuals were used to reduce genetic diversity, which can be a source of inter-individual variability in larval lake sturgeon development [46], and can affect gut microbiome composition and diversity [47]. Gamete collection and egg fertilization methods were as described in [39] and [44], respectively. Egg and free embryo stages were raised under the same conditions until the feeding experiment began. Fertilized eggs were maintained in Aquatic Eco-systems (Pentair, Inc., Delevan, WI, USA) J32 Mini-Egg hatching jars using 50-micron filtered stream water during incubation. Egg mortalities were monitored daily and removed. Upon hatching (9 May 2015), yolk sac fry were moved to 3.0 L polycarbonate aquaria (Pentair Aquatic Ecosystems, Cary, NC, USA). At eight days post-hatch (dph), free embryos were assigned randomly to six 3.0 L aquaria at a density of fifty larvae per tank [44].

The feeding transition experiment occurred over a five-week (36 days) time frame. At nine dph, aquaria were randomly assigned to two treatments: control (CR) and treated (TR), with three replicates each (*n* = 6 experimental units). Larvae in the CR treatment were fed live *Artemia* throughout the duration of the study (five weeks). Meanwhile, larvae in the TR treatment were fed *Artemia* nauplii during the first two weeks post exogenous feeding. Individuals of the TR treatment were then transitioned to frozen bloodworms (*Chironomus* spp., family Chironomidae) during week three of post exogenous feeding (21 dpf). On the initial transition day, TR fish were fed 90% dry body weight brine shrimp and 10% dry body weight bloodworms. On subsequent days, ratios of dry body weight for *Artemia* and bloodworms were as follows: day 2, 80% and 20%; day 3, 60% and 40%; day 4, 50% and 50%; day 5, 40% and 60%; day 6, 30% and 70%; and day 7, 15% and 85%, respectively. During week four until the end of the experiment, all individuals in the TR treatment were fed only bloodworms. The feeding regime was formulated based on [44,48] where the amount of food offered varied by week. In weeks 1 and 2, 26% dry body weight was offered; weeks 3 and 4, 13% dry body weight; and week 5, 11% dry body weight. Amount of *Artemia* and bloodworms were calculated based on established relationships between wet and dry weights of diets. Wet weight of *Artemia* was calculated from dry body weight at dry weight = 0.1767 (sieved wet weight) − 0.0541 [44]. Wet weight of bloodworms was calculated from dry weight at dry weight = 0.0832 (wet weight) + 0.0239 (Scribner, unpublished data). Because diets were offered in amounts suggested by previous research [48], feed intake was not monitored throughout the study. To promote feeding and maintain consistency, lake sturgeon were fed an equal amount of food three times daily (0900, 1300, and 1700) while water remained flowing, simulating stream flow. Brine shrimp cysts, Great Salt Lake strain, were purchased from Brine Shrimp Direct© (Ogden, UT, USA) and were cultured following manufacturer’s protocols. Frozen one-inch bloodworms were purchased from Brine Shrimp Direct©. Bloodworms were rinsed with ground water and mildly chopped using a blender before feeding.

Survival and growth were monitored throughout the transition experiment. Beginning 9 dph, mortalities were removed and recorded daily per aquarium. Larvae were weighed by replicate once per week to measure growth, and to alter the amount of food allocated to each aquarium in the following week. All larvae were collected in a dip net and water was removed by drying the net on a paper towel before weighing.

### 2.3. Morphometric Data and Survival Analyses

We quantified larval body size (total body length (TL) and body weight) as a function of time (sampling periods 14, 21, 36 days post exogenous feeding (dpf)), as a function of feeding cessation/transition treatment (control (CR) vs. treatment (TR)), and interactions between sampling time and treatment. Each of the 3.0-L aquaria represented an experimental unit. Mean weight (g ± SD) and TL (mm ± SD) for three replicates for each treatment (CR vs. TR) at each sampling time were estimated. While larval TL was measured based on three tank replicates at three sampling points, larval body weight was based on three replicates of each feeding treatments measured weekly throughout experimental duration. All statistical analyses were conducted using programming and statistical software, R (version v3.0.2; Amplitude, Inc., SanFransico, CA, USA). A mixed model ANOVA was used to analyze differences in measured TL and body weight per fish since the repeated measurements were taken across several time points. Specifically, we compared the mean differences of TL and body weight between two feeding treatments (CR vs. TR; i.e., “feeding” is a “between-subjects” factor) and over sampling times (dpf; “time” was the “within-subject” factor). A *p*-value ≤ 0.05 was considered statistically significant.

Survival was estimated as the mean daily proportion of larvae surviving from week 0 through week 5 (35 dpf) in program R using the survival package [49]. Survival curves were generated using the R package *survfit* function. Proportional survival was analyzed using a two-sample log-rank test using the R package *survdiff* function. The function was based on a chi-square distribution that quantified whether fish from different feeding treatments originated from the same or different distributions.

### 2.4. GI Tract Collection

Sampling of GI tracts occurred at three ontogenetic stages of development: two weeks post-exogenous feeding (14 dpf), three weeks (21 dpf), and five weeks (36 dpf). Prior to sampling, food was not administered for 18 h. Five larvae were sampled from each replicate and treatment for microbial community interrogation at each of the three stages. Fish were euthanized with an overdose of MS-222. Individuals were subsequently placed in microcentrifuge tubes and stored at room temperature in 80% ethanol for <2 mo. Given the small sizes of early-stage larvae, the ethanol was expected to help with fixation of the GI tract microbial communities. During each sampling period, hatchery water samples were also collected to document temporal variation in water microbial community diversity and taxonomic composition as a source of GI tract microbial colonization [50]. For water sampling, 500 mL of stream water was filtered using a 0.22 µm filter membrane (Sterlitech^®^, Auburn, WA, USA) using a hand-pump. The membrane was placed in a 50 mL tube and preserved with 80% ethanol. At the end of experimental duration, an additional five fish were collected per replicate and treatment and anesthetized with MS-222 (Sigma-Aldrich, St. Louis, MO, USA) in a petri dish. Fish were digitally photographed with a ruler to obtain total length before being preserved in liquid nitrogen for proteolytic bacteria studies (see Section 2.5 below). Digital images were analyzed using Image J software (NIH Image). All fish and water samples were stored until dissection and bacterial DNA extraction was performed within two months.

### 2.5. Fish Dissection

The distal GI tract (spiral valve) of each lake sturgeon larva was recovered following aseptic techniques. The spiral valve serves as the primary region of digestion and absorptive function, and represents an area of abundant nutrients where a microbial community can flourish [51,52]. Exterior surfaces were swabbed with 95% ethanol before dissections of the whole digestive tract using sterile instruments. Dissections were performed with slight modification as previously described by [53]. The intact alimentary tracts were cut out from the fish body cavities, and the whole excised GI tract was immediately transferred into filtered–sterilized 80% ethanol solution for DNA isolation. Due to the small size of each larval GI tract, a composite of four samples were grouped for each replicate (i.e., aquarium) within CR and TR treatments at each for each sampling period.

### 2.6. DNA Extraction of GI Tract Bacteria and 16S rRNA Amplicon Sequencing

Gastrointestinal tract microbiota from lake sturgeon larvae were surveyed using high-throughput sequencing of the V4 region of the 16S rRNA gene. Following dissection, tubes containing GI tract samples were first centrifuged for 15 min at 4 °C to pellet bacteria. The pellet was then washed and transferred into buffer. The MoBio PowerSoil^®^ DNA Isolation Kit (Qiagen, Inc., Carlsbad, CA, USA) was used to extract DNA following a bead-beating step following protocols for low-biomass samples, as suggested by the manufacturer. The integrity of each DNA sample was assessed based on amplification of 1.4 kbp of the 16S rRNA gene with primers 27F (AGAGTTTGATYMTGGCTCAG) and 1492R (ACGGGCGGTGTGTACAAG) followed by gel agarose electrophoresis and ethidium bromide staining. DNA concentrations were quantified using a Microplate spectrophotometer (BioTek^®^, Winooski, VT, USA).

Twenty-seven DNA samples (including two control samples) were sequenced at the Michigan State University Research Technology Support Facility (RTSF; East Lansing, MI, USA; (https://rtsf.natsci.msu.edu/genomics/; accessed on 1 June 2017). All sequencing procedures, including the construction of the Illumina sequencing library, emulsion PCR, and MiSeq paired-end (~250 bp; primer 515F and 806R) [54] followed standard Illumina (San Diego, CA, USA) protocols. The RTSF provided standard Illumina quality control, including base calling by Illumina Real Time Analysis v1.18.61, demultiplexing, adaptor and barcode removal, and RTA conversion to FastQ format by Illumina Bcl2Fastq v1.8.4 (Illumina, Inc., SanDiago, CA, USA). Raw sequence reads were deposited to NCBA Sequence Reads Archive (SRA) under Bioproject accession number PRJNA826657 (https://www.ncbi.nlm.nih.gov/sra/PRJNA826657).

### 2.7. Sequence Processing

Sequence data were processed using the default sequencing data analyses pipeline and computing workflow implemented by program Mothur v.1.42 [55] following the suggested settings of Mothur’s protocols (https://www.mothur.org/wiki/MiSeq_SOP). Briefly, paired-end sequence merging, quality filtering, “denoising”, singleton-sequence removal, chimera checking, and taxonomic assignments were conducted based on methods in Mothur as well as reference-based OTU clustering (method = opticluster). Taxonomic assignment was performed by first aligning sequences data using the SILVA 132 bacterial reference database followed by clustering sequences defined with 97% identity and later classified using Ribosomal Database Project (RDP) 16 (V5.7) training set. Given the short sequence length, OTU criteria representing sequences that are not more than 3% different from each other, and our desire to compare data presented here to previous gut microbiome research (e.g., [50]), we chose to define taxonomic variation based on OTUs rather than ASVs. Any sequence singletons that were detected were removed prior to downstream analyses. Rarefaction analyses were performed to evaluate the sampling coverage for each sample based on selected sequence depth. Final OTUs were rarefied to a depth of 9151 sequences per sample. Two DNA samples with low sequence depth were discarded in downstream analyses. The GitHub and DOI information for the repository of the community matrix of sequence counts by OTU and feeding treatment and sampling times are available at our GitHub site at GitHub: https://github.com/ScribnerLab/Compositional-dynamics-of-gastrointestinal-tract-microbiomes.git and DOI: https://doi.org/10.5281/zenodo.6901417 (accessed on 17 August 2022).

### 2.8. Isolation of Bacterial Culture and Extracellular Protease Screening

At the end of the feeding experiment, four fish were sampled from a replicate randomly chosen from each of the treatment (CR and TR). Fish were euthanized as described above and immediately dissected following the aseptic dissection procedure previously described. The freshly dissected GI tract was transferred to a tube containing 10 mL of sterile Luria Bertani (LB) broth and transported to Michigan State University for further processing. To prepare the intestinal homogenate, GI tracts from two larvae were transferred into separate microcentrifuge tubes, containing 250 µL of sterile LB. The GI tracts were subsequently homogenized using a sterile glass rod.

Homogenate samples were serially diluted in sterile LB up to 1:10^8^. An aliquot of 0.1 mL of each dilution was plated on each of three replicate plates of nutrient LB agar. Plates were incubated at 25 °C for 48–72 h. Following incubation, all colonies were counted, and their morphologies were documented. Each isolated colony was sub-cultured in a microtiter plate well containing 150 µL sterile LB media, incubated overnight, and stored at −80 °C in 15% glycerol concentration as a stock bacterial culture for further use.

Isolates were subjected to a plate assays to screen for extracellular microbial protease activities [56]. Milk protein agar plates were prepared in duplicates as follows: 20% (*w*/*v*) skim milk solution using casein powder and 2x purified LB agar was prepared separately in deionized water and autoclaved at 121 °C for 15 min. The milk solution was then added to agar to give a final concentration of 10% (*w*/*v*) of milk protein agar and 1x LB. The mixture was cooled to 55 °C before poured into plates [56]. Isolates were revived from storage in microtiter plates by defrosting on ice and then inoculating into a fresh microtiter plate with 150 µL LB broth per well using 96 solid pin multi-blot replicator. After incubation of the plate overnight, the strains were replicated onto LB agar supplemented with milk protein and incubated at room temperature for the first 24 h and transferred into 30 °C for an additional 24 h.

The zone of clearing around an inoculated strain indicated positive protease activity (clear morphs scored as either 0 as negative for protease and 1 as positive for protease). The clearing phenotype was recorded and photographed. Aliquots of LB containing each putative protease-producing isolate were subjected to Sanger sequencing for the16S rRNA gene using the 27F primer (AGAGTTTGATYMTGGCTCAG) at the RTSF.

### 2.9. Extracellular Protease Microbes in CR and TR Group

Sanger sequencing data were analyzed using Ribosomal Database Project (RDP, www.rdp.cme.msu.edu) (Michigan State University, East Lansing, MI, USA) pipeline (accessed on 10 January 2018). Sequences were first converted into FASTA format and manually trimmed to remove ambiguous bases. Taxonomic assignment was performed using SeqMatch in RDP. Simpson’s diversity index (SDI) of genus-level was estimated for each sample using vegan (version 2.4-2) package in R [57].

The 16S rRNA sequences were then aligned using ClustalW [58]. The phylogenetic relationship among all protease-producing bacteria isolated from the gut from fish of both CR and TR groups was inferred using a maximum-likelihood reconstruction method with the substitution model selected according to the Bayesian information criteria (BIC). The phylogenetic analysis was performed in MEGAX [59] using the general time reversible (GTR) model with bootstrap support of 500 times with *Escherichia coli* as an outgroup.

### 2.10. Analyses of Bacterial Community Profiles and Ecological Statistical Analyses

#### 2.10.1. Alpha Diversity

All measures of microbial community diversity including inverse Simpson (1/D) diversity indices and OTUs richness of each sample were calculated from the sequence data within program Mothur. Both metrices of inverse Simpson and taxa richness were not normally distributed even after data transformation. To test for significant differences in diversity indices between larvae from CR and TR treatments across three different ontogenetic stages (14, 21, and 36 dpf), we employed model reformation approaches based on a generalized linear model (GLM) using suitable probability distributions (inverse Simpson = gamma distribution; richness = Poisson distribution) in R program v3.0.2 using *glm().* GLM methods have been shown to have high efficiency when estimating parameters, yielding interpretable estimates that also avoid transformation bias [60,61]. *p*-values < 0.05 indicate significance of the effect of variables on alpha diversity measures. Relative abundance and identity of all phyla in all fish gut and water-associated microbial communities across sampling times were determined using packages *dplyr* and *reshape2* in program R.

#### 2.10.2. Beta Diversity

We used several packages implemented in program R to estimate comparative beta (β) diversity measures and ecological statistics at the OTU level. Briefly, we used the vegan package [57] to generate Bray–Curtis (BC) distances characterizing differences in microbial community composition among samples from different sampling times (dpf) and dietary treatments (TR and CR). We used the cmdscale function to perform principle coordinate analyses (PCoA) ordination of community composition differences based on inter-sample BC distances [62]. The ggplot and ggplots2 packages [63] were then used to create ordination plots to visually compare GI tract community composition with aquatic community composition as a function of different treatments and among sampling periods based on the principal coordinate with the three largest eigenvalues.

Multivariate hypotheses testing was used to quantify differences in community composition among samples collected at different times and from different feeding treatments using the adonis function [57] in program R. Community compositional differences based on comparisons between locations of the centroids of sampling groups for treatments and time periods were based on permutational multivariate analyses of variance (PERMANOVA) using BC community resemblance matrices [64,65]. Under the null hypotheses, food treatments were not expected to be significantly associated with fish GI tract microbial community taxonomic composition. This test was employed because of the non-parametric and skewed nature of microbial community data. OTU scores along the PCoA axes with the three largest eigenvalues (i.e., eigenvector associated with corresponding eigenvalue) were correlated with OTU scores along each original variable’s axis (i.e., relative proportion of measured taxa/OTU) to measure the OTUs’ contribution to a given PCoA axis [66] using the corr function.

### 2.11. Inferring Function of Members of GI Tract-associated Bacterial Communities

The 16S rRNA sequencing data and reference genomics databases were used to predict the functional roles of GI tract microbiota associated with larvae from TR and CR dietary treatments using program PICRUSt (version 1.1.0) [67] focusing at level 2 (function defined based on molecular interactions, protein post-translational modifications). We imputed the putative function of lake sturgeon GI tract using microbial taxonomic catalogues of annotated genes (Kyoto Encyclopedia of Genes and Genomes; KEGG) sequence database. With PICRUSt, we calculated a nearest sequenced taxon index (NSTI), which measures how closely related the average 16S rRNA sequence in the sample was to a sequenced genome. When this index is low (<0.05), PICRUSt is likely to perform well in predicting the genomes of the organisms in an environmental sample. High scores (>0.15) indicate few related references were available and prediction was of lower quality [67].

Reference-based OTU clustering and taxonomic assignments were performed using selected marker gene identifiers in the Greengenes database and converted into a biom file. The file was transferred into the PICRUSt program. Functional predictions were carried out based on information of the relative abundances of OTUs from larvae from the TR and CR diet treatments established from our 16S rRNA survey using an evolutionary model. The OTU table for each sample with associated Greengenes identifiers was later normalized based on the organism’s predicted 16S gene copy number using normalize_by_copy_number.py script. Functional roles were predicted by searching for pre-calculated genome content for each OTU using predict_metagenomes.py script. Annotation of predicted function was applied and summarized using the KEGG Orthology (KO) classification schemes using the categorize_by_function.py script, all included in PICRUSt. The program generated a profile table of annotated gene functions along with its abundance for each sample in the OTU table [67] to provide baseline to infer the functional attributes of observed taxa. In addition, PICRUSt calculated the nearest sequenced taxon index (NSTI) to quantify dissimilarity between reference genomes and the predicted metagenome presented here. The graphical representation and Welch’s t-test were both performed by STAMP [68] in program R to quantify significance of differences in mean proportion of sequence associated with predicted functions.

## 3. Results

### 3.1. Growth and Survival

**Figure 1:**
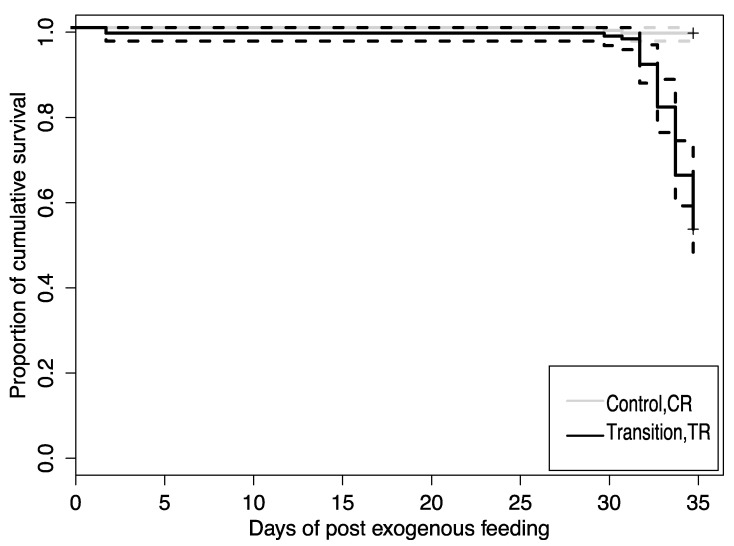
Mean cumulative survival (%; solid line) of lake sturgeon larvae from transition (TR) and control (CR) treatments with 95% CI (dashed line). Survival analyses were based on log-rank tests indicating that survival of fish in the TR treatment (0.527) was significantly lower compared to fish from the CR treatment (0.987) (chi-square value, χ^2^ = 85.1, *p*-value < 0.01, df = 1).

The proportion of fish surviving in each diet treatment at the end of the experiment (36 dpf) differed between dietary treatments (Figure 1). Larval lake sturgeon survival in the TR treatment (transition from *Artemia* to bloodworms) was significantly lower with nearly 50% mortality relative to fish in the CR treatment, of which 100% survived to the end of the 5-week study (chi-square value, χ^2^ = 85.1, *p*-value < 0.01, df = 1). The daily mortality of larvae in the TR dietary treatment peaked from 30 to 36 dpf.

The experiment was terminated after 36 days due to poor health of the larvae from the TR treatment. Collectively, a lack of growth (TL and weight) and a lower survival of larvae from the dietary treatment TR vs. CR was attributed to decreasing amounts of *Artemia* relative to blood worms in the TR dietary treatment during diet transition and ultimately cessation of feeding and starvation.

**Figure 2:**
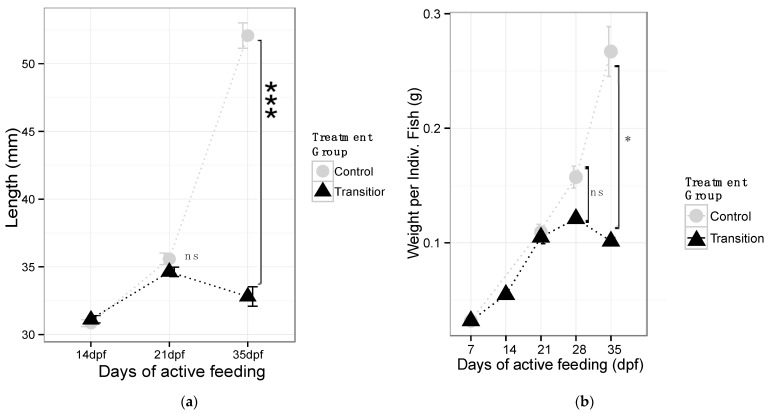
Larval lake sturgeon morphometric data. (**a**) Mean total length (mm) by treatment, at each sampling point; (**b**) average body weight (g) of larvae from each feeding treatment. Significant differences in treatment mean test based on a Welch-*t* test (ns indicates non-significant; * *p*-value < 0.05; *** *p*-value < 0.001).

Analyses of morphometric data, (TL and weight; Figure 2a,b) were based on comparisons of mean weight (g ± SD) and mean total length (TL; mm ± SD) between larvae from different dietary treatments and across time (dpf). There was a significant interaction between the sampling time and the dietary treatment associated with the mean TL (df = 2, F = 99.62, *p*-value < 0.0001, Supplementary Table S1). At 14 days post-feeding (dpf) and 21 dpf, no significant difference was detected between the mean TL from fish in both treatments (14 dpf: CR 30.84 ± 1.00 mm, TR 31.13 ± 1.05 mm, *p*-value = 0.66; 21 dpf: CR 35.59 ± 1.65 mm, TR 34.63 ± 1.36 mm, *p*-value = 0.32). However, at the end of the experiment (36 dpf), the mean TL for fish from the TR treatment (33.15 ± 2.36 mm) were significantly lower (*p*-value < 0.001) than fish from the CR treatment (52.08 ± 3.64 mm; Figure 2a).

A significant interaction was found between the sampling time and the dietary treatment associated with body weight (df = 3, F = 111.5, *p* < 0.0001, Table 1b). At the end of the experiment, larvae in the TR dietary treatment had a lower weight than larvae in the CR dietary treatment (36 dpf: CR 0.267 ± 0.04 g, TR 0.101 ± 0.01 g, *p*-value < 0.05, *p*-value < 0.01) whereas for 21 dpf and 28 dpf, no significant differences were observed (CR-0.111 ± 0.01 g, TR-0.105 ± 0.01 g, *p*-value = 0.459, Figure 2b). Measurements at the first and second week were not included due to the lack of data from all replicates.

### 3.2. Characterization of Diversity and Taxonomic Composition of Microbial GI Tract Communities

#### 3.2.1. Estimates of Community Alpha Diversity

**Figure 3:**
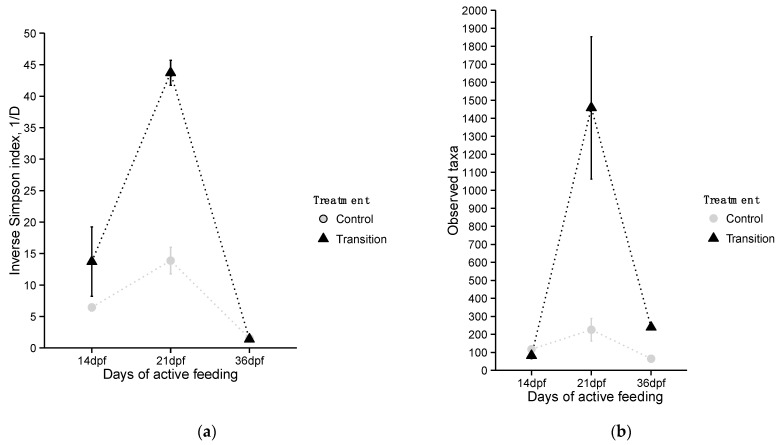
Measures of microbial community diversity including (**a**) inverse Simpson diversity index and (**b**) number of observed OTUs (taxa richness) for larval lake sturgeon from the control (CR) and transition (TR) feeding treatments at different sampling times.

Measures of GI tract microbial community diversity at the OTU level including the inverse Simpson index and taxa richness (number of taxa detected) indicated differences in diversity between treatments across sampling times (dpf; Figure 3a,b). GLM (Supplementary Table S2) indicated TR treatment had significantly influenced both diversity indices (inverse Simpson: *p*-value < 0.05; taxa richness: *p*-value < 0.001). Findings also indicate that alpha diversity indices of larvae from diet treatments differed significantly during the last two sampling periods (inverse Simpson: *p*-value_21dpf_ = 0.002, *p*-value_36dpf_ < 0.001; taxa richness: *p*-value_21dpf_ < 0.001, *p*-value_36dpf_ < 0.001).

#### 3.2.2. Estimates of Community Composition at the Phyla Level

**Figure 4:**
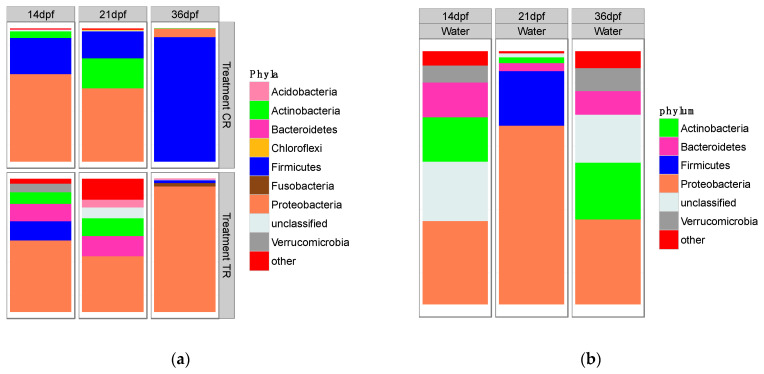
(**a**) Relative abundance (%) of six most abundant bacterial phyla (*Acidobacteria*, *Actinobacteria*, *Bacteroidetes*, *Firmicutes*, *Fusobacteria*, and *Proteobacteria*) found in the larval lake sturgeon GI tract for each treatment at different sampling times. The remaining taxa were assigned as others. (**b**) Relative abundance (%) of six most abundant bacterial phyla (*Acidobacteria*, *Actinobacteria*, *Bacteroidetes*, *Firmicutes*, *Proteobacteria*, and *Verrucomicrobia*) found in aquatic samples. The remaining taxa were assigned as others.

At 14 dpf, GI tract microbial community samples of twenty-four fish from all six replicates in CR and TR dietary treatments indicated that at the level of phyla, six phyla were present in both treatments. Phyla differed in relative abundance between feeding treatments. However, all phyla were well represented, including more than 90% of total GI tract microbial taxonomic composition; *Proteobacteria* (mean TR 53.7%, mean CR 65.6%), *Firmicutes* (mean TR 14.5%, mean CR 27.1%), *Actinobacteria* (mean TR 8.8%, mean CR 5.1%), *Acidobacteria* (mean TR 0.05%, mean CR 0.39%), *Bacteroidetes* (mean TR 12.9%, mean CR 0.4%), and *Verrucomicrobia* (mean TR 6.5%, mean CR 0.2%). The difference in mean proportions of these phyla is shown in Figure 4a (and Supplementary Table S3). Each feeding treatment also had three unique phyla present in low proportions (~ 1% or less; Supplementary Table S4).

During the attempted transition to bloodworms during week 3 (21 dpf), we observed a difference in microbial community composition between the two dietary treatment groups. Greater numbers of phyla were detected in TR larval samples (*Chlorobi*, *Fusobacteria*, *Lentisphaerae*, *Nitrospira*, *Parcubacteria (OD1)*, *Microgenomates (OP11)*, *Absconditabacteria (SR1)*, *Synergistetes*, *Saccharibacteria (TM7)*). These phyla were absent in control larvae (CR) fed only *Artemia* (Supplementary Table S3. We also observed an increased abundance of *Acidobacteria* (mean relative abundance increased from less than 1% to 5.9%) and unclassified phyla (mean relative abundance increased from 2.5% to 8.1%) as the experiment continued from 14 dpf to 21 dpf in the TR treatment. In larvae from the TR feeding treatment, we documented declines in abundance of *Firmicutes* following the transition week (14 dpf: 15%, 21 dpf: 6%), while *Firmicutes* proportions remained relatively stable in CR treatment (14 dpf: 27.2%, 21 dpf: 20.2%). After the transition week at 21 dpf, the GI tract community composition for larvae in the CR treatment was dominated by three major phyla (*Firmicutes*, *Actinobacteria*, and *Proteobacteria)* totaling more than 95% of the sequences. At the end of the experiment (36 dpf), the GI tract microbial communities from larvae in both CR and TR treatments were distinct. The *Firmicutes* phylum dominated the community composition of larvae in the CR dietary treatment (93.5%), while GI tract communities of TR dietary treatment larvae were dominated by *Proteobacteria* (94.3%). Overall patterns of phyla present in the larvae GI tract contrasted greatly with phyla detected in water samples, during all three sampling periods (Figure 4b).

#### 3.2.3. Estimates of Beta Diversity: Associations between GI Tract Microbial Community Composition and Feeding Treatments, across Sampling Periods

**Figure 5:**
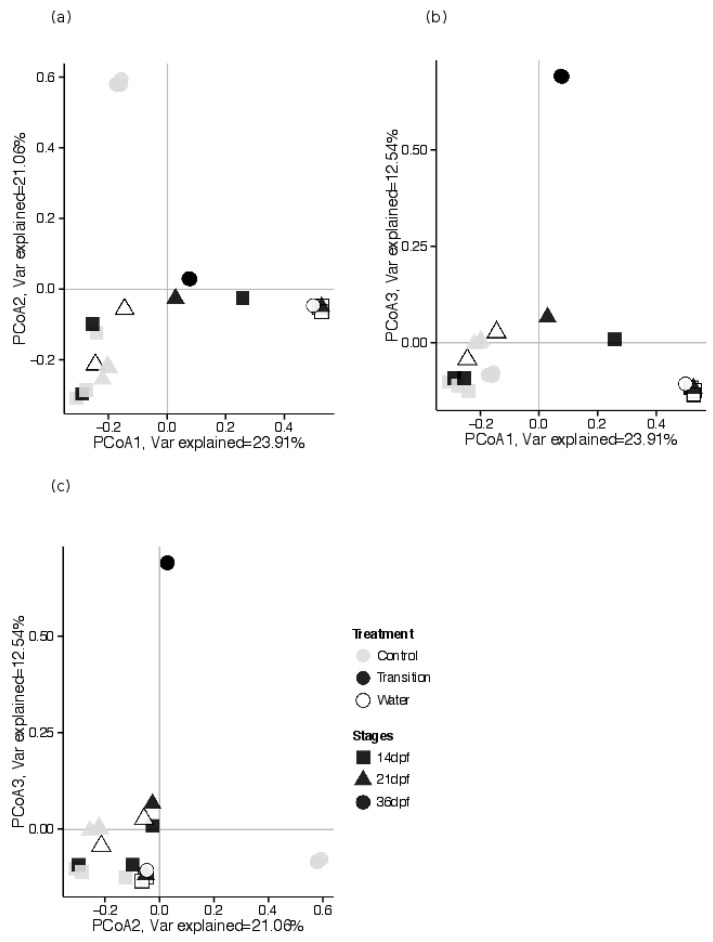
Visualization of measures of microbial community beta diversity (Bray–Curtis distance) based on multivariate principal coordinates analyses (PCoA) plots showing variation in larval lake sturgeon GI tracts and water microbial community compositional differences among collections made at different times and between control (CR) and transition (TR) food treatments. Samples were taken from replicates of different treatments at three developmental stages (before transition at 14d pf, during transition week at 21 dpf, after transition at 36 dpf). (**a**) Plot based on Bray–Curtis distance axis 1 and 2, (**b**) plot based on Bray–Curtis distance axis 1 and 3, (**c**) plot based on Bray–Curtis distance axis 2 and 3.

Microbial taxonomic composition and relative abundance differed significantly by dietary treatment and sampling period (PERMANOVA test, pseudo-F = 2.928, R^2^ = 0.151, *p* < 0.001, df = 2; Table 2). Microbial community composition of larvae from both CR and TR treatments were similar prior to diet transition (14 dpf). Considerable overlap in GI tract community compositions and water was documented during early sampling periods in larvae from the CR treatment as well as the TR treatment (Figure 5a–c). Subsequently, GI tract communities from larvae in the TR dietary treatment started to diverge from those of larvae in the CR dietary treatment as these communities clustered along axes PCo 1 and 3 (21 dpf, Figure 5b,c). Larvae from the CR dietary treatment showed higher taxonomic compositional similarity across sampling times compared to TR dietary treatment larvae that were characterized by large compositional heterogeneity during 36 dpf as communities disperse across axes 2 and 3 (Figure 5a,c).

We further quantified the feeding treatment effects of diet across the sampling times (dpf) on community composition at the OTU level using linear regression and least square mean analyses. As expected, no significant effect of treatment was detected at 14 dpf, (F-statistics = 1.184, *p*-value: 0.338). As diets of larvae in the TR group transitioned from brine shrimp to bloodworms and feeding levels decreased at 21 dpf, we observed a significant difference between both dietary treatments (F-statistics = 140.2, *p*-value < 0.01). Divergence in community taxonomic composition continued through the end of the experiment at 36 dpf (F-statistics = 1.213 × 10^5^, *p*-value < 0.001; Table 2b).

PCoA axes-1 of all three sampling periods explained the most variation in microbial communities representing the linear combination of all taxonomic composition.

Correlation analyses of the relative abundance of observed taxa (OTUs) with PCoA associated with the three largest eigenvalues found 17 taxa that highly correlated (Pearson correlation > 0.70) with those eigenvectors (Table 3). A total of 14 OTUs show strong, positive correlation with the first principal coordinate axis (see Table 3 for list of OTUs). In concordance with the divergence between microbial communities in fish from CR and TR treatment as they reach 36 dpf, two OTUs from phylum *Firmicutes* Otu001 (genus *Clostridium_sensu_stricto*) and Otu10 (unclassified *Clostridiceae*) were strong, positively correlated with the second principal coordinate (Pearson correlation = 0.94; 0.87). This is a match with the farther distance of CR fish microbial communities from the origin in the direction along the PCo2-axis (Figure 5c). In contrast, Otu2 (genus *Aeromonas*, phylum *Proteobacteria*) was strongly correlated (Pearson correlation = 0.97) with the third principal coordinate (PCo3-axis). Along this axis, gut microbial communities of TR fish located farther than the origin compared to CR fish, indicating that taxonomical compositions in TR fish larvae are strongly driven by the presence of this taxa.

### 3.3. Predicted Functional Roles of Lake Sturgeon Larval GI Tract Microbiota

Functional inventories of microbiome genes, such as those involved in the metabolism of macronutrients (carbohydrates, amino acid, and lipids) were predicted from bacterial species assemblages using PICRUSt. Estimated NSTI across all larval GI tract samples show that this metric fell within the range of 0.048 to 0.258 (mean 0.22).

**Figure 6:**
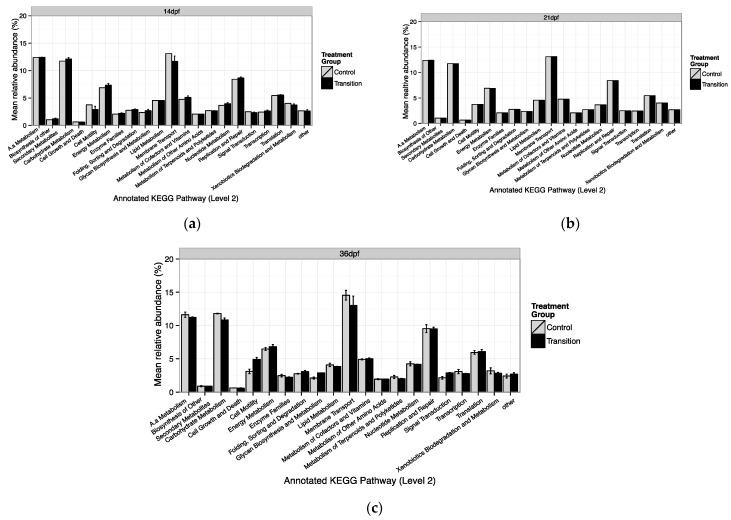
Relative abundance of the 20 most prevalent annotated functional groups identified using KEGG orthologs pathway categorized at molecular-level 2 associated with lake sturgeon larval GI tract microbiome associated with two food treatments (control (CR) and transition (TR)) at each sampling point (**a**) 14 dpf; (**b**) 21 dpf; and (**c**) 36 dpf.

The program inferred 37 gene families from the GI tract samples of larvae from both feeding treatments across sampling points (Figure 6a–c; Supplementary Table S4). Of these 37 gene families, the majority of the genes were associated with membrane transport (mean value across sampling periods 13.58 ± 0.84% in CR-associated communities and 12.62 ± 0.81 in TR-associated communities, respectively), carbohydrate metabolism (11.75 ± 0.03% in CR, 11.56 ± 0.66% in TR), amino acid (12.13 ± 0.45% in CR, 12.01 ± 0.70% in TR), replication and repair (8.78 ± 0.64% in CR, 7.01 ± 0.29% in TR), and energy metabolism (6.75 ± 0.25% in CR, 7.01 ± 0.29% in TR). The mean relative abundances of these predicted gene families are relatively stable across time. None of the predicted 37 gene families varied significantly in abundance between treatments.

### 3.4. Activity-Based Screening for Protease-Positive Isolates

The screening for bacterial isolates that exhibited proteolytic activity was based on the presence of clearing zones on skimmed-milk agar among pure colonies isolated from GI tract samples. Proteolytic assays resulted in the detection of a total of 140 isolates from the GI tract of fish larvae from both the CR and TR treatments. Out of these, Sanger sequencing data were retained for 54 bacterial isolates obtained from TR and 44 sequences from CR during RDP sequence alignment and taxonomic identification.

**Figure 7:**
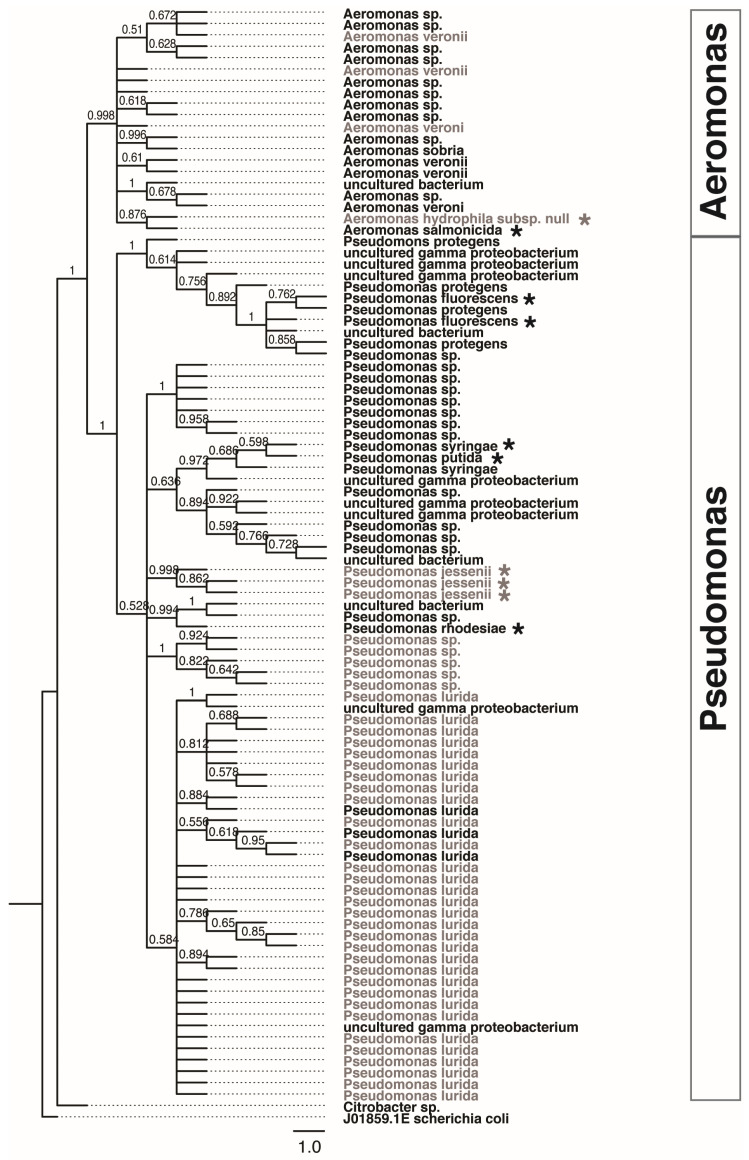
Distribution of protease-producing strains isolated from the gut of lake sturgeon fed different diet treatments. Larvae in control (CR) group were fed with live *Artemia nauplii* whereas treated (TR) group were fed with *Artemia* nauplii during the first 14 days post feedings (dpf) before transitioned to commercially grown bloodworm at 21 dpf. Isolates in bold text are microbial communities found larvae in the treated (TR) group and the remaining were from CR group. The maximum-likelihood phylogenetic tree was constructed using 16S rRNA sequence in MEGAX with general time reversible model and bootstrap support of 500 times. Protease-producers in both treatments were identified as *Pseudomonas* and *Aeromonas* with an isolate of *Citrobacter* sp. was identified in the control group. Pseudomonads and Aeromonads marked with an asterisk are species uniquely found in the TR group, and several are known to be associated with stress-related diseases in various fish species.

Phylogenetic relationships of these identified strains isolated from fish in CR and TR treatments were inferred using the maximum likelihood (ML) tree (Figure 7). Most isolates originated from the genera *Aeromonas* and *Pseudomonas*. OTUs shown in the tree were based on the top matches sequence similarity score in RDP, values range between 0.91 and 0.958, representing considerable phylogenetic breadth across species.

We observed the higher SDI values in proteolytic bacterial isolates present in larvae from the TR compared to the CR treatment (0.533 in TR vs. 0.165 in CR). While no specific grouping for the *Aeromonas* bacteria was observed between the two experimental groups, there was an unequal distribution of the *Pseudomonas* bacteria between larvae from the CR and TR groups. In particular, three clades that were identified belong to bacteria isolated from the fish with the transitional bloodworm diet. Unique isolates for the TR group clustered in these clades (*Pseudomonas fluroscens*, *P. putida*, *P. protegens*, and *P. rhodesiae*), and were marked with asterisks (Figure 7). Only one clade associated with CR was found to contain a unique cluster of *Pseudomonas jessenii*. Two *Aeromonas* species were found to be present only in TR treatment individuals (*A. salmonicida*) and CR (*A. hydrophila*) fish, respectively, yet both clustered in the same clade.

## 4. Discussion

This study characterized growth and survival changes as well as taxonomic compositional and phylogenetic relationships of larval lake sturgeon GI tract microbial communities that were sampled from two feeding treatments across larval ontogeny. We found that larval lake sturgeon associated with the TR treatment that had ceased feeding showed reduced growth and survival and exhibited phylogenetically distinct GI tract microbial communities at the end of the experiment (36 dpf). The phylum *Proteobacteria (Pseudomonadota)* dominated GI tract microbial communities of larvae from the TR treatment whereas larvae in the CR dietary treatment were dominated by *Firmicutes (Bacillota);* however, the diversity of GI tract microbial communities in fish larvae were significantly different between treatments, potentially associated with recurrent colonization from ambient river water microbial communities or due to the mobilization of substances in the GI tract in response to feeding cessation that favored a phylogenetically different suite of taxa. Importantly, this study demonstrates that the disparities in GI tract microbiota of apparently healthy (CR treatment) compared to starved (TR treatment) lake sturgeon larvae may provide fish health metrics to inform aquaculturalists and microbiologists of normal vs. aberrant GI tract development.

### 4.1. Larval Growth and Survival

Larval lake sturgeon failed to transition from brine shrimp to bloodworms leading to poor growth and survival. Visual inspections of larval stomachs indicated that TR treatment larvae did not eat bloodworms, and that consumption had ceased by 21 dpf when brine shrimp were no longer provided as a portion of the diet. A lack of ingestion of bloodworms by individuals in the TR dietary treatment likely explains significant differences in the mean total length, mean weight, and survival compared to larvae in CR treatment. We suspect that at the development stage of larvae when the transition started, frozen bloodworms were inadequate to stimulate sensory organs and feeding response. Studies have shown that live *Artemia* provide visual stimuli and excrete free amino acid metabolites that serve as feeding stimuli, contributing to greater acceptance in fish larvae [13,69]. In our study, frozen bloodworms may not have provided the same visual and chemical stimuli, so feeding did not occur. Low survival and growth rates of TR treatment fish prompted the feeding trial to be terminated at 36 dpf. Thus, our GI tract microbial results may characterize communities in starved individuals rather than those undergoing ontogenetic food transitioning periods.

### 4.2. Characterization of Diversity and Taxonomic Composition of Microbial GI Tract Communities

GI tract community composition of larvae from the two treatments differed following dietary transition (i.e., at 21 dpf and 36 dpf, but not at 14 dpf). Prior to transitioning, we expected and documented that the GI tract microbial communities would be similar between treatments since larvae received the same diet. The divergence in community composition and diversity was observed as soon as bloodworms were introduced and feeding levels declined. This divergence could be attributed to both the differences in diets between treatment groups as well as a lack of food ingestion of TR treatment individuals. As an example of diets possibly affecting microbiota, *Artemia* are phagotrophic filter-feeders that can ingest food rapidly and are incubated in salt water, which indicates that *Artemia* could accumulate high bacterial loads and of a phylogenetically different taxonomic composition during the cultivation process that could later be transferred to GI tracts of fish larvae after consumption [70]. It is possible that microbial communities inside the GI tract of larvae in the CR treatment were influenced by microbes present in *Artemia* during the latter half of the study, forming a consortium of microbes distinct from larvae in the TR treatment. However, we believe the lack of food ingestion more profoundly affected the microbial communities between the two treatments. Thus, microbial community differences between treatments should reflect those of starved (TR) and healthy (CR) larvae, and these communities may inform culturalists of impending morbidity and mortality or health of reared larvae.

Larval lake sturgeon that ceased feeding had a higher taxonomic diversity of GI tract microbes than those that were feeding. However, the composition of the bacteria community changed and was dominated by members of one bacterial group: *Proteobacteria* (or recently revised as *Pseudomonadota*). Previous studies parallel our results where alterations occur in the GI tract community composition of individuals that experience prolonged fasting or starvation. Starvation in zebrafish over 21 days lead to increased diversity and altered microbial composition that showed relative increases in *Vibrio* (phylum *Proteobacteria/Pseudomonadota*) [71]. In other aquaculture species, including Nile tilapia (*Oreochromis niloticus*) and Asian seabass (*Lates calcarifer*), the relative abundance of *Proteobacteria* also increased in prolonged fasting individuals compared to well-nourished individuals [36,72]. In human gut microbiota studies, members of Proteobacteria are believed to indicate dysbiosis. This broad group usually is in low abundance in the gut of a healthy human and has been linked to obesity and diabetes [73,74].

Results from human studies are similar to our findings of large increases in the relative abundance of *Aeromonas* in the GI tract communities of TR larvae. These changes may occur because during the fasting, the supply of nutrients to GI tract symbionts were significantly reduced, causing an “energy crisis” [36,75], where some microbial species may not be able to survive. Further, other studies show that starved fish show evidence of intracellular degradation and reduced endothelium that subsequently reduced the tissue mass of the host digestive system, and in turn could have affected the taxonomic composition of microbiota [36,72].

Gastrointestinal tract microbial communities may have differed between larvae from the two treatments due to the inherent microbial communities associated with the food sources. Although fish from the TR treatment likely experienced starvation, the presence of bloodworm in the surrounding water might have affected the aquatic pool of microbial taxa that could have colonized the gut tracks of TR fish larvae. Chironomids were found as natural reservoirs of *Vibrio cholerae* and *Aeromonas* spp. [76,77]. Fish are frequently recorded as feeding on chironomids. Thus, they might be acquiring these microbes via chironomid consumption.

Community compositional differences between treatments indicate that the diet and stress may enrich for functionally different microbial communities. PICRUSt analyses were conducted to predict functional pathway properties that were consistent with the taxonomic compositional variation identified qualitatively (e.g., using PCoA ordination; Figure 5) [67]. Functional group composition of GI tract microbes was generally consistent between treatments throughout the study. The most abundant functional categories identified were associated with carbohydrate metabolism, energy metabolism, amino acid metabolism, membrane transport and replication and repair. Results were consistent with general metabolic functions (e.g., carbohydrate, protein and amino acid metabolism) essential for microbial survival [78]. In mammals, the GI tract microbiota play an important role in aiding the supply of alternative energy sources, such as ketone bodies, when hosts are faced with fasting and starvation [35,79]. Studies by Xia et al. [80] reported similar categories of enriched taxa in Asian seabass (*Lates calcarifer*) following starvation. Taxa in that study were in functional categories including carbohydrate transport and metabolism, inorganic ion transport and metabolism, and amino acid transport and metabolism. A study on marine ascidia based on microbiome characterizations and metabolomics also reported that biosynthesis and metabolic pathways involving the gut microbiome were upregulated, suggesting a beneficial contribution of the gut microbiome to the host under stress [81].

Responses of microbial taxa to fasting have been shown to vary across hosts and by functional group of microbial taxa [80]. Our results show significant taxonomic divergence was attributed to a reduction in the prevalence of three bacterial functional groups involved in transcription, cell division and chromosome partitioning, replication, as well as recombination and repair, while another five functional groups (membrane transport, carbohydrate metabolism, energy metabolism, amino acid metabolism, replication, and repair) increased in prevalence. Sullam et al. [82] also reported similar findings when they performed multiple comparisons between difference ecotypes of Trinidadian guppies *(Poecilia reticulata)*. Nonetheless, almost all functional categories differed when compared across enterotypes. As a caveat, our results should be reviewed with caution as predictions of gene families to functional groups using PICRUSt had high NSTI metrics. A high NSTI value indicates high discrepancies between our 16S metagenome dataset and reference genomes [67], and these probably attributed to a lack of marine or freshwater animal PICRUSt reference microbial genome that limits te resolution of functional prediction.

GI tract community composition and diversity in fishes is tied to diet [83]. The host GI tract is composed of taxonomically diverse suites of species, many of which have mutualistic relationships with the host, including the production of secondary metabolites [45]. The role of protein fermentation by proteolytic microorganisms is particularly important to host health and digestive proficiency, including the conversion of carbohydrates to beneficial short chain fatty acids [45]. Thus, we investigated the presence of proteolytic bacteria, with the aim to see a possible involvement in the digestion of a protein-rich diet. Protease-producing bacterial taxa found in larval lake sturgeon GI tracts from both TR and CR feeding treatments consisted mostly of taxa from the genera *Pseudomonas* or *Aeromonas*, a greater abundance of these genera were detected in fish from the TR dietary treatment. While we cannot rule out the possibility that culture-based screening methods could lead to bias in determining bacterial numerical dominance, the inflated numbers of these two genera could be attributed to the abundance of these bacterial taxa in the fish-surrounding (river water) environment. These bacteria may have been present in the aquarium water, contributing to their presence in the GI tracts of fish in both treatments. *Pseudomonas* are also commonly part of fish intestinal and egg microbial communities [84,85].

*Aeromonas* is commonly isolated from aquatic environments and from clinical tissue samples from humans or animals, and have also been shown to be present in chironomids and brine shrimp [77,86]. In addition, numbers of *Aeromonas* sp. were regarded not only as an important disease-causing pathogen in poikilothermic animals, but were also identified as etiologic agents causing intestinal illnesses in humans [87]. Species such as *A. hydrophila* ATCC 7966, and *A. salmonicida* cause great loss in commercially important salmonids in aquacultural facilities. The ability to produce extracellular protease is one of the virulence factors possessed by these species associated with pathogenicity. Thus, the presence of *Pseudomonas* and *Aeromonas* in higher abundance within the GI tract of TR treatment (starved) lake sturgeon may be a factor leading to or resulting from intestinal dysbiosis.

The hypothesis of diversity resistance assumes that a species with a more diverse microbial community has a greater antagonistic potential against invaders or pathogens [88]. Our data contradicts this hypothesis as reflected by the higher SDI values in larvae from the TR treatment compared to the CR treatment (0.533 in TR vs. 0.165 in CR). Fish that failed to transition to bloodworms exhibited greater proteolytic bacterial taxonomic diversity. While no unique clustering for the *Aeromonas* bacteria was observed between the two treatment groups, there was a remarkably high distribution of the *Pseudomonas* bacteria in TR treatment larvae. Two Pseudomonads clustered in three clades within the TR treatment larvae GI tracts, namely *P. putida*, *P. protegens,* and *P. fluorescence*. These Pseudomonads have been described as the opportunistic pathogens that cause serious stress-related diseases in aquaculture industries of various fishes [89,90,91]. Thus, the larvae that had ceased feeding hosted microbiota indicative of diseased individuals, which may have exacerbated mortality. A greater abundance of Pseudomonads in fish that ceased feeding may indicate pathogenic colonization as several *Pseudomonas* species are known plant and animal pathogens [92].

Ontogenetic dietary changes may be associated with the colonization of microbes that lead to GI tract inflammation. As an example, a recent study observed the enrichment of several genera that could be associated with GI tract inflammation leading to issues such as diarrhea as well as a reduction of several beneficial bacterial families in post-weaned piglets [93]. As a second example, many factors including dietary changes and stresses have impacted the homeostasis of intestinal microbial communities that increase the susceptibility to bowel inflammation [94]. Thus, the introduction of stresses via dietary changes potentially weaken the individual by increasing the inflammation and permeability level of the gut, although the mechanistic understanding on the interplay between diet, microbiome, and disease susceptibility remains to be explored.

Given the importance of GI tract microbial communities and the potential application of advanced microbial-based strategies in the artificial rearing of larval lake sturgeon and fish in general, more in depth studies are warranted to further characterize the genotypic and functional diversity of these communities. Future studies could focus on how microbial communities change during and after successful dietary transitions in cultured fishes as well as how specific diets affect the microbial community composition. It would be important to know which diet regimes support beneficial gut microbes versus which ones benefit less desirable microbes. The efficiency and quality of live prey should also be evaluated and carefully monitored, especially during the first six weeks of offering exogenous feed to larvae to ensure health and maximum growth.

## 5. Conclusions

This work provided an in-depth characterization of changes in GI tract microbial taxonomic composition and diversity over early fish ontogenetic developmental stages in response to feeding cessation following a failed diet transition. While not an intended outcome, the data provide a microbial signature of nutritionally and metabolically induced dysbiosis at the community level. This dysbiosis is most likely in response to feeding cessation and was coupled with the reduced growth and high mortality of the larvae. Potentially pathogenic bacterial communities dominated with the GI tracts of starved fish. Combined, these data are of importance to fisheries managers and microbiologists generally of diagnostic GI tract community membership and diversity in fishes that are tied to diet-induced dysbiosis.

## Figures and Tables

**Table 1:** Split-plot ANOVA (mixed design ANOVA) table for larval lake sturgeon. (**a**) Total length; (**b**) mean larval body weight indicate sources of variability between-tank replicate associated with feeding treatment and within-tank replicate associated with sampling times. A significant interaction was also observed between treatment and stages.

(**a**)					
**Source**	**df**	**SS**	**MS**	**F**	***p*-Value**
**Between subject (tank) effect**					
Trt treatment	1	191.94	191.94	64.41	0.001
Error	4	11.92	2.98		
**Within subject (tank) effect**					
Time	2	416.90	208.44	119.63	<0.0001
Time*Trt	2	347.10	173.57	99.62	<0.0001
Residuals	8	13.90	1.74		
(**b**)					
**Source**	**df**	**SS**	**MS**	**F**	***p*-Value**
**Between-subject (tank) effect**					
Trt treatment	1	0.1208	0.1208	121.8	<0.001
Error	4	0.0040	0.0010		
**Within-subject (tank) effect**					
Week	3	0.1057	0.0352	101.5	<0.0001
Week*Trt	3	0.1161	0.0387	111.5	<0.0001
Residuals	12	0.0042	0.0004		

**Table 2:** (**a**) PERMANOVA analysis quantifying sources of variability among larval lake sturgeon GI tract microbiota community composition that differed significantly among sampling times and between treatments. A significant interaction was also observed between treatment and sampling times (PERMANOVA test pseudo-F = 2.928, R^2^ = 0.087, *p* < 0.001; permutation = 1000). (**b**) Goodness of fit from linear regression model (R^2^), *p*-value, and least-square means analyses performed on significantly important PCo axes calculated separately for each sampling period. PCo axis 1 for period 21 dpf and PCo axis 1 for period 36 dpf showed significant differences across diet treatment influencing the microbial community composition, but none of PCo axis for period 14 dpf was found to be significant.

(**a**)						
	**Df**	**Sum Sq**	**Mean Sq**	**F-Value**	**R^2^**	**Pr (>F)**
**Stages**	3	2.710	0.903	3.518	0.273	<0.001
**Treatment**	1	1.106	1.106	4.308	0.111	<0.001
**Stages:Treatment**	2	1.504	0.752	2.928	0.151	<0.001
**Residuals**	18	4.623	0.257		0.465	
(**b**)						
**Linear regression model:** **Pco Axes~Treatment**				**Least square means**		
**Important axes**		**Estimates**		***p*** **-value**	**Control**	**Transitioned**
**PCo1 14 dpf (Before transition)**		0.321		0.338	−0.160 ± 0.209	0.160 ± 0.209
**PCo1 21 dpf (Transition week)**		−0.754		<0.01	0.302 ± 0.040	−0.453 ± 0.049
**PCo1 36 dpf (After transition)**		−0.990		<0.001	0.330 ± 0.002	−0.660 ± 0.002

**Table 3:** List of microbial taxa with established taxonomic identification that were shown to be highly correlated with eigenvectors of the first three principal coordinate axes associated with largest eigenvalues.

PCo. Axes	Taxa	Taxonomic Identification/ Genera	Correlation
**2**	Otu1	*Clostidium_sensu* *stricto*	0.94
**3**	Otu2	*Aeromonas*	0.97
**2**	Otu10	Unclassified *Clostridiceae*	0.87
**1**	Otu11	Unclassified *Betaproteobacteria*	0.92
**1**	Otu12	Unclassfied *Microbacteriaceae*	0.84
**1**	Otu15	Unclassified *Comamonadeceae*	0.88
**1**	Otu16	Unclassified *Actinomycetales*	0.86
**1**	Otu17	Unclassified *Comamonadeceae*	0.83
**1**	Otu18	Unclassified *Comamonadeceae*	0.88
**1**	Otu19	*Polynucleobacter*	0.89
**1**	Otu24	Unclassified *Actinomycetales*	0.88
**1**	Otu29	Unclassified *Sphingobacteriales*	0.88
**1**	Otu31	Unclassified *Cryomorphaceae*	0.898
**1**	Otu41	Unclassified *Microbacteriaceae*	0.83
**1**	Otu42	*Methylophilus*	0.78
**1**	Otu45	Unclassified *Cytophagaceae*	0.88
**1**	Otu48	Unclassified *Sphingomonadaceae*	0.74

## Data Availability

Raw sequence data have been deposited in NCBI accession number: PRJNA826657. The OTU community matrix of sequence counts by OTU and treatment sample can be found on GitHub at GitHub: GitHub: https://github.com/ScribnerLab/Compositional-dynamics-of-gastrointestinal-tract-microbiomes.git (accessed on 17 August 2022) and DOI: https://doi.org/10.5281/zenodo.6901417 (accessed on 17 August 2022).

## Supplementary Materials

The following supporting information can be downloaded at: https://www.mdpi.com/article/10.3390/microorganisms10091872/s1, Table S1. Comparisons between total length of larvae from both CR and TR feeding treatments using Welch t-test at each sampling point. Table S2. Results from generalized linear models (GLM) in measures of microbial community diversity. (a) Inverse Simpson; (b) OTU richness among fish fed with control brine shrimp diet (CR) vs. fish transitioned from brine shrimp to bloodworm diet (TR) across three different ontogenetic stages (14, 21, and 36 dpf). Table S3. Comparison of the phyla in GI tract samples of fish from CR and TR. Mean proportion were calculated across tank replicate. ND indicate that the phylum was not detected. Table S4. Predicted functions of the GI tract-associated microbial communities between fish from the CR vs. TR treatment across stages. Differentially abundant of these functional genes were tested by Welch-*t* test comparison at each sampling stage. All means was not significant based on adjusted *p*-value using false discovery rate method (FDR).

## Declarations

Ethics and approval to participate.
