# Supplementary material for: Compositional Dynamics of Gastrointestinal Tract Microbiomes Associated with Dietary Transition and Feeding Cessation in Lake Sturgeon Larvae
Source: Microorganisms. 2022 Sep 19;10(9):1872. doi: 10.3390/microorganisms10091872 (PMC9500890; doi:10.3390/microorganisms10091872)
Supplement: Supplementary file 1 [file microorganisms-10-01872-s001.zip › Suppl Table S1.pdf]

Table S1 Comparisons between total length of larvae from both CR and TR feeding treatments using Welch t-test at each sampling point.

14dpf

Source	df	SS	MS	F	p-value
**Trt**	1	0.139	0.139	0.246	0.646
**Residuals**	4	2.178	0.545		
**Total**	5				

21dpf

Source	df	SS	MS	F	p-value
**Trt**	1	1.384	1.384	1.276	0.322
**Residuals**	4	4.340	1.085		
**Total**	5				

36dpf

Source	df	SS	MS	F	p-value
**Trt**	1	537.56	537.56	111.18	0.00046***
**Residuals**	4	19.34	4.83		
**Total**	5				
