# Supplementary material for: Compositional Dynamics of Gastrointestinal Tract Microbiomes Associated with Dietary Transition and Feeding Cessation in Lake Sturgeon Larvae
Source: Microorganisms. 2022 Sep 19;10(9):1872. doi: 10.3390/microorganisms10091872 (PMC9500890; doi:10.3390/microorganisms10091872)
Supplement: Supplementary file 1 [file microorganisms-10-01872-s001.zip › Suppl Table S2.pdf]

Table S2. Results from Generalized Linear Models (GLM) in measures of microbial community diversity (a) Inverse Simpson; (b) OTU richness among fish fed with control brine shrimp diet (**CR**) vs fish transitioned from brine shrimp to bloodworm diet (**TR**) across three different ontogenetic stages (**14**, **21**, and **36 dpf**)

(a) Inverse Simpson

Model: Inverse Simpson ~ Treatment + Stages

Coefficients	Estimate	Standard Error	t-value	Pr (>t)
**Intercept**	0.134	0.017	8.002	< 0.001***
**Treatments Transition**	-0.024	0.014	-3.940	0.0017**
**Stages 21dpf**	-0.038	0.014	-3.994	0.0015**
**Stages 36dpf**	-0.085	0.093	6.015	< 0.001***

Null deviance: 22.277, df 16
Residual deviance: 1.337, df 13
Dispersion parameter for Gamma is 0.112

(b) OTUs richness

Model: OTUs richness ~ Treatment + Stages

Coefficients	Estimate	Standard Error	z-value	Pr (>z)
**Intercept**	3.638	0.049	74.796	< 0.001***
**Treatments Transition**	1.446	0.033	44.478	<0.001***
**Stages 21dpf**	2.108	0.044	47.664	<0.001***
**Stages 36dpf**	0.440	0.055	7.972	< 0.001***

Null deviance: 7227.83, df 16
Residual deviance: 931.49, df 13
Dispersion parameter for Poisson is 1
