# Supplementary material for: Compositional Dynamics of Gastrointestinal Tract Microbiomes Associated with Dietary Transition and Feeding Cessation in Lake Sturgeon Larvae
Source: Microorganisms. 2022 Sep 19;10(9):1872. doi: 10.3390/microorganisms10091872 (PMC9500890; doi:10.3390/microorganisms10091872)
Supplement: Supplementary file 1 [file microorganisms-10-01872-s001.zip › Suppl Table S3.pdf]

Table S3. Comparison of the phyla in GI tract samples of fish from CR and TR. Mean proportion were calculated across tank replicate. ND indicate that the phylum was not detected.

Phyla	Stages	Proportion by Treatment	
		CR	TR
**Acidobacteria**	14dpf	0.386%	0.047%
**Actinobacteria**	14dpf	5.121%	8.808%
**Armatimonadetes**	14dpf	ND	0.892%
**Bacteroidetes**	14dpf	0.357%	12.935%
**Chlamydiae**	14dpf	ND	0.007%
**Chloroflexi**	14dpf	ND	0.022%
**Firmicutes**	14dpf	27.174%	14.512%
**Fusobacteria**	14dpf	0.004%	ND
**OP11**	14dpf	0.004%	ND
**Planctomycetes**	14dpf	0.091%	ND
**Proteobacteria**	14dpf	65.599%	53.703%
**Verrucomicrobia**	14dpf	0.193%	6.487%
**unclassified**	14dpf	1.071%	2.586%
**Phyla**	**Stages**	**CR**	**TR**
**Acidobacteria**	21dpf	0.175%	5.912%
**Actinobacteria**	21dpf	22.613%	13.370%
**Armatimonadetes**	21dpf	0.040%	0.863%
**Bacteroidetes**	21dpf	0.062%	15.255%
**Chlamydiae**	21dpf	0.113%	0.071%
**Chlorobi**	21dpf	ND	0.033%
**Chloroflexi**	21dpf	0.543%	0.301%
**Fusobacteria**	21dpf	ND	3.590%
**Firmicutes**	21dpf	20.224%	5.508%
**Gemmatimonadetes**	21dpf	0.011%	1.027%

**Lentisphaerae**	21dpf	ND	0.005%
**Nitrospira**	21dpf	ND	0.098%
**OD1**	21dpf	ND	0.175%
**OP11**	21dpf	ND	0.055%
**Planctomycetes**	21dpf	0.433%	0.301%
**Proteobacteria**	21dpf	54.992%	41.788%
**SR1**	21dpf	ND	0.022%
**Synergistetes**	21dpf	ND	0.153%
**TM7**	21dpf	0.015%	0.038%
**Verrucomicrobia**	21dpf	0.124%	3.344%
**unclassified**	21dpf	0.656%	8.092%
**Phyla**	**Stages**	**CR**	**TR**
**Acidobacteria**	36dpf	0.014%	ND
**Actinobacteria**	36dpf	0.344%	0.082%
**Bacteroidetes**	36dpf	0.011%	0.388%
**Firmicutes**	36dpf	93.536%	1.962%
**Fusobacteria**	36dpf	ND	2.595%
**OD1**	36dpf	ND	0.005%
**Proteobacteria**	36dpf	6.049%	94.328%
**Verrucomicrobia**	36dpf	0.003%	ND
**unclassified**	36dpf	0.044%	0.639%
