# Supplementary material for: Compositional Dynamics of Gastrointestinal Tract Microbiomes Associated with Dietary Transition and Feeding Cessation in Lake Sturgeon Larvae
Source: Microorganisms. 2022 Sep 19;10(9):1872. doi: 10.3390/microorganisms10091872 (PMC9500890; doi:10.3390/microorganisms10091872)
Supplement: Supplementary file 1 [file microorganisms-10-01872-s001.zip › Suppl Table S4.pdf]

Table S4. Predicted functions of the GI tract-associated microbial communities between fish from the CR vs TR treatment across stages. Differentially abundant of these functional genes were tested by Welch-t test comparison at each sampling stage. All means was not significant based on adjusted p-value using False Discovery Rate method (FDR).

Stages	Level_2	CR Mean rel. abund. (%)	CR Std. dev	CR SEM	TR Mean rel. abund. (%)	TR Std. dev	TR SEM	Diff. betw. means	95% lower CI	95% upper CI	p-values	FDR
**14dpf**	Amino Acid Metabolism	12.391	0.000	0.000	12.434	0.044	0.031	-0.044	-0.597	0.510	0.500	0.501
**14dpf**	Biosynthesis of Other Secondary Metabolites	1.043	0.000	0.000	1.210	0.167	0.118	-0.167	-2.284	1.951	0.500	0.501
**14dpf**	Cancers	0.164	0.000	0.000	0.161	0.003	0.002	0.003	-0.038	0.045	0.500	0.501
**14dpf**	Carbohydrate Metabolism	11.737	0.000	0.000	12.119	0.382	0.270	-0.382	-5.238	4.474	0.500	0.501
**14dpf**	Cardiovascular Diseases	0.016	0.000	0.000	0.010	0.007	0.005	0.007	-0.080	0.094	0.500	0.501
**14dpf**	Cell Communication	0.001	0.000	0.000	0.000	0.000	0.000	0.000	-0.003	0.004	0.500	0.501
**14dpf**	Cell Growth and Death	0.653	0.000	0.000	0.609	0.044	0.031	0.044	-0.516	0.604	0.500	0.501
**14dpf**	Cell Motility	3.750	0.000	0.000	2.901	0.849	0.601	0.849	-9.943	11.642	0.500	0.501
**14dpf**	Circulatory System	0.046	0.000	0.000	0.029	0.017	0.012	0.017	-0.198	0.232	0.500	0.501
**14dpf**	Digestive System	0.035	0.000	0.000	0.047	0.012	0.009	-0.012	-0.167	0.143	0.500	0.501
**14dpf**	Endocrine System	0.590	0.000	0.000	0.525	0.065	0.046	0.065	-0.763	0.893	0.500	0.501
**14dpf**	Energy Metabolism	6.896	0.000	0.000	7.334	0.438	0.309	-0.438	-5.997	5.122	0.500	0.501
**14dpf**	Environmental Adaptation	0.163	0.000	0.000	0.163	0.000	0.000	0.000	-0.004	0.004	0.495	0.501
**14dpf**	Enzyme Families	2.083	0.000	0.000	2.219	0.136	0.096	-0.136	-1.861	1.589	0.500	0.501
**14dpf**	Excretory System	0.031	0.000	0.000	0.045	0.014	0.010	-0.014	-0.186	0.159	0.500	0.501

**14dpf**	Folding, Sorting and Degradation	2.762	0.000	0.000	2.894	0.132	0.093	-0.132	-1.810	1.546	0.500	0.501
**14dpf**	Glycan Biosynthesis and Metabolism	2.354	0.000	0.000	2.687	0.334	0.236	-0.334	-4.572	3.905	0.500	0.501
**14dpf**	Immune System	0.055	0.000	0.000	0.063	0.009	0.006	-0.009	-0.119	0.101	0.500	0.501
**14dpf**	Immune System Diseases	0.041	0.000	0.000	0.049	0.008	0.006	-0.008	-0.114	0.097	0.500	0.501
**14dpf**	Infectious Diseases	0.442	0.000	0.000	0.439	0.003	0.002	0.003	-0.035	0.041	0.500	0.501
**14dpf**	Lipid Metabolism	4.565	0.000	0.000	4.545	0.020	0.014	0.020	-0.236	0.277	0.500	0.501
**14dpf**	Membrane Transport	13.087	0.001	0.001	11.681	1.405	0.994	1.406	-16.452	19.263	0.500	0.501
**14dpf**	Metabolic Diseases	0.104	0.000	0.000	0.111	0.007	0.005	-0.007	-0.097	0.083	0.500	0.501
**14dpf**	Metabolism of Cofactors and Vitamins	4.758	0.000	0.000	5.116	0.357	0.253	-0.357	-4.895	4.180	0.500	0.501
**14dpf**	Metabolism of Other Amino Acids	2.075	0.000	0.000	2.056	0.019	0.014	0.019	-0.226	0.265	0.500	0.501
**14dpf**	Metabolism of Terpenoids and Polyketides	2.699	0.000	0.000	2.681	0.018	0.013	0.018	-0.210	0.245	0.501	0.501
**14dpf**	Nervous System	0.088	0.000	0.000	0.091	0.003	0.002	-0.003	-0.046	0.039	0.500	0.501
**14dpf**	Neurodegenerative Diseases	0.336	0.000	0.000	0.266	0.070	0.049	0.070	-0.819	0.958	0.500	0.501
**14dpf**	Nucleotide Metabolism	3.655	0.000	0.000	3.950	0.295	0.209	-0.295	-4.043	3.453	0.500	0.501
**14dpf**	Replication and Repair	8.416	0.000	0.000	8.669	0.253	0.179	-0.253	-3.467	2.961	0.500	0.501
**14dpf**	Sensory System	0.000	0.000	0.000	0.000	0.000	0.000	0.000	-0.001	0.001	0.500	0.501

**14dpf**	Signal Transduction	2.495	0.000	0.000	2.332	0.164	0.116	0.164	-1.917	2.244	0.500	0.501
**14dpf**	Signaling Molecules and Interaction	0.147	0.000	0.000	0.195	0.049	0.034	-0.049	-0.665	0.568	0.500	0.501
**14dpf**	Transcription	2.434	0.000	0.000	2.642	0.208	0.147	-0.208	-2.853	2.437	0.500	0.501
**14dpf**	Translation	5.454	0.000	0.000	5.544	0.089	0.063	-0.090	-1.225	1.046	0.499	0.501
**14dpf**	Transport and Catabolism	0.422	0.000	0.000	0.451	0.028	0.020	-0.028	-0.387	0.330	0.500	0.501
**14dpf**	Xenobiotics Biodegradation and Metabolism	4.012	0.000	0.000	3.733	0.279	0.197	0.279	-3.262	3.819	0.500	0.501
**21dpf**	Amino Acid Metabolism	12.388	0.003	0.002	12.398	0.000	0.000	-0.009	-0.046	0.028	0.199	0.351
**21dpf**	Biosynthesis of Other Secondary Metabolites	1.043	0.001	0.000	1.046	0.000	0.000	-0.003	-0.010	0.004	0.131	0.348
**21dpf**	Cancers	0.164	0.000	0.000	0.164	0.000	0.000	0.000	-0.002	0.001	0.175	0.351
**21dpf**	Carbohydrate Metabolism	11.736	0.000	0.000	11.744	0.000	0.000	-0.008	-0.010	-0.006	0.006	0.057
**21dpf**	Cardiovascular Diseases	0.016	0.000	0.000	0.017	0.000	0.000	0.000	-0.001	0.001	0.304	0.433
**21dpf**	Cell Communication	0.001	0.000	0.000	0.001	0.000	0.000	0.000	0.000	0.000	0.218	0.351
**21dpf**	Cell Growth and Death	0.653	0.000	0.000	0.652	0.000	0.000	0.000	-0.002	0.003	0.331	0.433
**21dpf**	Cell Motility	3.748	0.000	0.000	3.734	0.000	0.000	0.014	0.011	0.018	0.008	0.059
**21dpf**	Circulatory System	0.046	0.000	0.000	0.046	0.000	0.000	0.000	-0.001	0.001	0.678	0.737
**21dpf**	Digestive System	0.035	0.000	0.000	0.034	0.000	0.000	0.000	0.000	0.000	0.131	0.348
**21dpf**	Endocrine System	0.589	0.000	0.000	0.588	0.000	0.000	0.001	-0.004	0.005	0.372	0.451
**21dpf**	Energy Metabolism	6.894	0.000	0.000	6.884	0.000	0.000	0.010	0.005	0.015	0.025	0.133

**21dpf**	Environmental Adaptation	0.163	0.000	0.000	0.163	0.000	0.000	0.000	0.000	0.001	0.132	0.348
**21dpf**	Enzyme Families	2.084	0.001	0.001	2.082	0.000	0.000	0.002	-0.012	0.016	0.323	0.433
**21dpf**	Excretory System	0.031	0.000	0.000	0.032	0.000	0.000	0.000	0.000	0.000	0.001	0.035
**21dpf**	Folding, Sorting and Degradation	2.762	0.000	0.000	2.758	0.000	0.000	0.004	-0.001	0.009	0.058	0.237
**21dpf**	Glycan Biosynthesis and Metabolism	2.353	0.000	0.000	2.347	0.000	0.000	0.006	0.004	0.008	0.015	0.093
**21dpf**	Immune System	0.055	0.000	0.000	0.055	0.000	0.000	0.000	-0.001	0.000	0.144	0.351
**21dpf**	Immune System Diseases	0.041	0.000	0.000	0.041	0.000	0.000	0.000	-0.001	0.001	0.948	0.960
**21dpf**	Infectious Diseases	0.443	0.000	0.000	0.442	0.000	0.000	0.000	-0.001	0.002	0.200	0.351
**21dpf**	Lipid Metabolism	4.563	0.002	0.001	4.561	0.000	0.000	0.002	-0.018	0.022	0.378	0.451
**21dpf**	Membrane Transport	13.097	0.001	0.000	13.143	0.001	0.001	-0.046	-0.054	-0.039	0.003	0.040
**21dpf**	Metabolic Diseases	0.104	0.000	0.000	0.104	0.000	0.000	0.000	0.000	0.000	0.079	0.293
**21dpf**	Metabolism of Cofactors and Vitamins	4.759	0.001	0.001	4.755	0.000	0.000	0.004	-0.008	0.016	0.154	0.351
**21dpf**	Metabolism of Other Amino Acids	2.075	0.001	0.000	2.076	0.000	0.000	-0.001	-0.008	0.005	0.291	0.433
**21dpf**	Metabolism of Terpenoids and Polyketides	2.697	0.001	0.001	2.696	0.000	0.000	0.001	-0.015	0.017	0.591	0.684
**21dpf**	Nervous System	0.088	0.000	0.000	0.088	0.000	0.000	-0.001	-0.001	0.000	0.002	0.040
**21dpf**	Neurodegenerative Diseases	0.336	0.000	0.000	0.336	0.000	0.000	0.000	-0.004	0.004	0.898	0.949

**21dpf**	Nucleotide Metabolism	3.657	0.002	0.001	3.653	0.000	0.000	0.003	-0.021	0.028	0.339	0.433
**21dpf**	Replication and Repair	8.418	0.004	0.003	8.406	0.000	0.000	0.012	-0.037	0.061	0.200	0.351
**21dpf**	Sensory System	0.000	0.000	0.000	0.000	0.000	0.000	0.000	0.000	0.000	0.218	0.351
**21dpf**	Signal Transduction	2.494	0.000	0.000	2.490	0.000	0.000	0.004	0.001	0.007	0.036	0.166
**21dpf**	Signaling Molecules and Interaction	0.147	0.000	0.000	0.147	0.000	0.000	0.000	-0.002	0.002	0.960	0.960
**21dpf**	Transcription	2.436	0.002	0.001	2.437	0.000	0.000	-0.001	-0.021	0.019	0.614	0.688
**21dpf**	Translation	5.454	0.002	0.001	5.443	0.000	0.000	0.012	-0.013	0.036	0.106	0.348
**21dpf**	Transport and Catabolism	0.422	0.000	0.000	0.421	0.000	0.000	0.001	-0.002	0.003	0.210	0.351
**21dpf**	Xenobiotics Biodegradation and Metabolism	4.009	0.004	0.003	4.016	0.000	0.000	-0.007	-0.055	0.042	0.336	0.433
**36dpf**	Amino Acid Metabolism	11.618	0.801	0.401	11.204	0.157	0.111	0.414	-1.006	1.834	0.449	0.824
**36dpf**	Biosynthesis of Other Secondary Metabolites	0.898	0.150	0.075	0.904	0.009	0.006	-0.006	-0.280	0.268	0.947	0.993
**36dpf**	Cancers	0.146	0.018	0.009	0.157	0.027	0.019	-0.011	-0.216	0.193	0.747	0.954
**36dpf**	Carbohydrate Metabolism	11.791	0.095	0.047	10.827	0.422	0.298	0.965	-4.038	5.967	0.258	0.824
**36dpf**	Cardiovascular Diseases	0.010	0.007	0.003	0.011	0.006	0.004	0.000	-0.028	0.028	0.993	0.993
**36dpf**	Cell Communication	0.000	0.000	0.000	0.000	0.000	0.000	0.000	0.000	0.001	0.182	0.824
**36dpf**	Cell Growth and Death	0.628	0.025	0.013	0.598	0.072	0.051	0.030	-0.756	0.817	0.748	0.954
**36dpf**	Cell Motility	3.092	0.674	0.337	4.910	0.414	0.293	-1.818	-3.694	0.058	0.054	0.465
**36dpf**	Circulatory System	0.026	0.020	0.010	0.087	0.023	0.016	-0.061	-0.206	0.083	0.171	0.824

**36dpf**	Digestive System	0.028	0.009	0.004	0.042	0.001	0.001	-0.014	-0.030	0.001	0.063	0.465
**36dpf**	Endocrine System	0.407	0.183	0.091	0.389	0.031	0.022	0.018	-0.307	0.343	0.879	0.956
**36dpf**	Energy Metabolism	6.461	0.434	0.217	6.804	0.469	0.331	-0.344	-3.251	2.564	0.598	0.851
**36dpf**	Environmental Adaptation	0.181	0.018	0.009	0.188	0.002	0.001	-0.008	-0.040	0.025	0.524	0.851
**36dpf**	Enzyme Families	2.456	0.379	0.190	2.221	0.073	0.051	0.235	-0.438	0.907	0.373	0.824
**36dpf**	Excretory System	0.041	0.010	0.005	0.019	0.003	0.002	0.022	0.004	0.040	0.027	0.465
**36dpf**	Folding, Sorting and Degradation	2.742	0.098	0.049	3.052	0.248	0.175	-0.310	-2.893	2.273	0.422	0.824
**36dpf**	Glycan Biosynthesis and Metabolism	2.098	0.264	0.132	2.859	0.030	0.021	-0.760	-1.236	-0.285	0.014	0.465
**36dpf**	Immune System	0.075	0.021	0.011	0.072	0.012	0.008	0.003	-0.050	0.056	0.864	0.956
**36dpf**	Immune System Diseases	0.067	0.027	0.013	0.050	0.012	0.008	0.017	-0.039	0.073	0.433	0.824
**36dpf**	Infectious Diseases	0.419	0.025	0.012	0.482	0.054	0.038	-0.063	-0.590	0.464	0.440	0.824
**36dpf**	Lipid Metabolism	4.078	0.506	0.253	3.821	0.056	0.040	0.257	-0.655	1.169	0.447	0.824
**36dpf**	Membrane Transport	14.550	1.455	0.727	13.024	1.973	1.395	1.525	-13.068	16.118	0.578	0.851
**36dpf**	Metabolic Diseases	0.103	0.002	0.001	0.095	0.005	0.003	0.008	-0.040	0.056	0.332	0.824
**36dpf**	Metabolism of Cofactors and Vitamins	4.909	0.160	0.080	4.993	0.183	0.129	-0.084	-1.267	1.100	0.733	0.954
**36dpf**	Metabolism of Other Amino Acids	1.942	0.143	0.071	1.926	0.024	0.017	0.017	-0.237	0.270	0.858	0.956
**36dpf**	Metabolism of Terpenoids and Polyketides	2.268	0.438	0.219	2.005	0.059	0.041	0.263	-0.522	1.048	0.379	0.824

**36dpf**	Nervous System	0.095	0.008	0.004	0.083	0.006	0.004	0.012	-0.017	0.041	0.237	0.824
**36dpf**	Neurodegenerative Diseases	0.258	0.079	0.039	0.475	0.091	0.064	-0.216	-0.807	0.374	0.204	0.824
**36dpf**	Nucleotide Metabolism	4.231	0.593	0.297	4.151	0.039	0.027	0.080	-1.002	1.162	0.831	0.956
**36dpf**	Replication and Repair	9.519	1.186	0.593	9.494	0.336	0.238	0.025	-2.106	2.157	0.975	0.993
**36dpf**	Sensory System	0.000	0.000	0.000	0.000	0.000	0.000	0.000	0.000	0.000	0.182	0.824
**36dpf**	Signal Transduction	2.135	0.365	0.182	2.863	0.114	0.081	-0.728	-1.394	-0.062	0.039	0.465
**36dpf**	Signaling Molecules and Interaction	0.216	0.069	0.035	0.172	0.014	0.010	0.044	-0.079	0.166	0.365	0.824
**36dpf**	Transcription	3.067	0.634	0.317	2.761	0.053	0.038	0.305	-0.846	1.457	0.468	0.824
**36dpf**	Translation	5.938	0.544	0.272	6.082	0.415	0.294	-0.144	-2.179	1.891	0.806	0.956
**36dpf**	Transport and Catabolism	0.322	0.101	0.050	0.358	0.023	0.016	-0.036	-0.215	0.143	0.598	0.851
**36dpf**	Xenobiotics Biodegradation and Metabolism	3.184	0.902	0.451	2.820	0.192	0.136	0.363	-1.234	1.960	0.551	0.851
